# Early father-infant skin-to-skin contact and its effect on the neurodevelopmental outcomes of moderately preterm infants in China: study protocol for a randomized controlled trial

**DOI:** 10.1186/s13063-018-3060-2

**Published:** 2018-12-22

**Authors:** Qingqi Deng, Qiufang Li, Hua Wang, Huilian Sun, Xinfen Xu

**Affiliations:** 1 Women’s Hospital, School of Medicine, Zhejiang University, Hangzhou, China; 2 Haining Maternal and Child Health Hospital, Branch of Women’s Hospital, School of Medicine, Zhejiang University, Hangzhou, China

**Keywords:** Skin-to-skin contact, Fathers, Preterm, Neurodevelopmental outcomes

## Abstract

**Background:**

Skin-to-skin contact (SSC) is an evidence-based intervention that benefits low birth weight /preterm infants. However, China’s health institutional policy inhibits parents from visiting their baby in the neonatal intensive care unit (NICU). In addition, the Chinese traditional postpartum behavioral practice of confining women to home raises barriers to mother-infant contact. Thus, to shorten the duration of parent-infant separation, father-infant SSC is considered a possible alternative. This study determines whether it is safe to perform father-infant SSC in the NICU and investigates how paternal SSC affects outcomes compared with traditional care (TC) for moderately preterm infants.

**Methods/design:**

A randomized controlled trial will be used to investigate the effects of paternal-infant SSC in NICU wards in China. Preterm infants born at a gestational age in the range of 32^0^–34^6^ weeks with a birth weight > 1500 g will be eligible. A simple random sampling method will be used to allocate infants to the SSC group (*n* = 25) or the TC group (*n* = 25). After medical stability, infants in the SSC group will be provided SSC by fathers for one hour every day until discharged from hospital. The primary outcome is neurodevelopmental measures, specifically salivary cortisol and Premature Infant Pain Profile (PIPP) during hospitalization. At 40 weeks of corrected age, infants will be assessed using the Infant Neurological International Battery (INFANIB) and neuroimaging. Secondary outcomes include infants’ physiological stability during SSC and throughout hospitalization and state observation at discharge. The fathers’ mental health will be assessed with the State-Trait Anxiety Inventory (STAI) 1 day to 3 days after the infant’s admission to the NICU and at discharge. Father-infant attachment will be evaluated at 4 and 6 months after the infants’ discharge, measured by the Paternal Postnatal Attachment Scale (PPAS). Statistical analyses will be conducted using a two-sided significance level of 0.05.

**Discussion:**

The effects of paternal-infant SSC on moderately preterm infants will be assessed. The data gathered in this study may have important implications for medical practice and policy in the NICU regarding the care methods of premature infants in China.

**Trial registration:**

Chinese Clinical Trial Registry, ChiCTR-IOR-1701274. Registered on 20 September 2017. Retrospectively registered.

**Electronic supplementary material:**

The online version of this article (10.1186/s13063-018-3060-2) contains supplementary material, which is available to authorized users.

## Background

Every year, approximately 15 million babies are born preterm (gestational age (GA) less than 37 weeks), and more than 60% of these preterm births occur in Africa and South Asia [1]. China ranks second in the top 10 countries with the greatest number of preterm deliveries [2]. According to the National Health and Family Planning Commission of the People’s Republic of China, preterm births account for approximately 7.0% of the total births in China, and they are the main cause of infant mortality [3]. Although the survival rate of preterm infants has increased because of the recent advances in neonatal care, premature births can cause varying degrees of adverse long-term neurodevelopmental impairment, which burdens both the family and country [4].

Preterm birth interrupts the normal process of brain maturation, putting infants at great risk of neurodevelopmental vulnerability and fragility [5]. During the third trimester, brain volume increases approximately 2.7-fold from 29 to 41 weeks postconception, which means that very preterm infants have a significantly smaller total brain volume than their term-born peers [6]. These reductions persist throughout childhood and adolescence [7]. Regional brain volumes near term are a promising marker for predicting disturbances in cognitive and behavioral outcomes in preterm infants [8]. Moreover, newborns must be exposed to the extrauterine environment in the neonatal intensive care unit (NICU) while the immature preterm brain is not yet ready to process various stimuli, including lights, noise, and painful interventions [9]. The infants’ sensory experience before term may cause negative effects on brain development and alter brain function and structure [10, 11].

Among those adverse stimuli, painful invasive procedures are the prominent experience that infants must undergo within the context of the NICU. Evidence shows that preterm infants experience a median of 10 painful procedures per day during hospitalization, 79.2% of which occur without specific analgesia [12, 13]. Unrelieved repeated pain exposure is associated with subsequent alterations in pain sensitivity, which may result in deleterious consequences including emotional, behavioral, and learning disabilities [14].

Nevertheless, preterm infants’ developmental outcomes appear to be modified by improving the NICU environmental experience and providing neuroprotective caregiving practices, as supported by the concept that the young brain has the ability of neuroplastic response [9]. This considerable plasticity of the brain can be expected at approximately 3 months before term age [15]. These critical and sensitive periods of brain development can create “windows of opportunity” for NICU-based interventions that may be beneficial for neurodevelopment [16].

Skin-to-skin contact, also known as kangaroo care (KC), is one of the recommended interventions to improve preterm birth outcomes according to the WHO [17]. SSC is globally accepted under different circumstances. In low-income settings, SSC is ideally provided for 24 h/day as a health care strategy; in affluent settings, SSC is considered an alternative option to shorten the length of parent-infant separation [18]. Regardless of how SSC is applied, multiple lines of evidence have suggested the short-term benefits of SSC for preterm infants. SSC has been used as a pain treatment to attenuate behavioral responses as well as to decrease cortisol levels. Studies found that Premature Infant Pain Profile (PIPP, a behavioral measure of pain for premature infants) scores and salivary and serum cortisol were lower on response to heel sticks in preterm infants who received SSC than in those who were treated with incubator care [19, 20].

Furthermore, infants who received SSC were found to have more organized sleep patterns, with longer periods in the alert wakefulness and quiet sleep states [21]. SSC infants showed a more mature neurodevelopmental profile [21, 22] and scored higher on the Bayley Scales of Infant Development and Psychomotor Developmental Index at 6 months [23]. In addition to these existing behavioral sleep findings, more rigorous neurophysiologic results measured by electroencephalographic/polysomnographic records also demonstrated that fewer rapid eye movements, more quiet sleep, and lower arousal were noted in SSC preterm infants than in the control cohort [24, 25], indicating the effect of SSC on improving sleep organization and accelerating brain maturation. Because sleep is vital for normal development, SSC may be used as an intervention to promote better sleep patterns within the context of the NICU environment. In addition to the above benefits, it is acknowledged that SSC helps establish parent-infant attachment.

According to the regulation theory of Schore [26], early maternal-infant separation can pose risk on attachment patterns, which is associated with alterations in brain structure and function. Research showed that very low birth weight (LBW) preterm infants with neurological impairment were more likely to develop an insecure quality of attachment [27]. Maternal-infant interactions were less synchronous at 3 months [28], and mothers of preterm infants were more likely to suffer from psychiatric illness (depression, anxiety) [29], which contributed to adverse attachment outcomes [30]. The physical closeness and sensory stimulations of SSC play an important role in regulating maternal-infant interaction. Evidence demonstrates that SSC can attenuate the negative psychological effects of premature birth by enhancing mothers’ sense of competence and sensitivity towards their infants, by decreasing mothers’ stress scores and increasing maternal-infant attachment scores [31–33], and by facilitating a better maternal-infant interactive style [34, 35].

Although it remains unclear whether SSC has long-lasting effects on preterm infants in terms of neurodevelopmental outcomes, studies show that KC may have a direct effect on infant neurophysiological organization [23]. Long-term follow-up studies have found that KC preterm infants show attenuated stress responses, have organized sleep and better cognitive control, and maintain long-lasting social and behavioral protective effects even after 10 and 20 years [36, 37].

KC has been recognized as an evidence-based intervention to improve health outcomes for LBW/preterm infants; however, it is not common in China. According to the national policy in China, parents are not allowed to enter the NICU to stay with their infants. Parent-infant separation begins after delivery until the infant is discharged, which accounts for a long period of time. This model of NICU care is accepted because it avoids the incidence of infection and also because of the ease of managing the patients. For severely ill infants, one of the preventive measures is to reduce the incidence of infection, which should be addressed by professionals. However, for medically stable preterm infants, a meta-analysis showed that LBW infants who received KC had reduced incidence of mortality, nosocomial infection, and severe illness compared to those who did not receive KC [38]. These results also support the idea that parents entering the NICU wards did not increase the risk of LBW infants becoming infected [38, 39]. Furthermore, the concept of family-centered care (FCC), which allows parents to participate in taking care of the baby, has been increasingly considered an important component of the NICU [40]. Evidence shows that FCC is feasible and safe, and it does not increase the rates of nosocomial infection; the incidence of necrotizing enterocolitis (NEC) was significantly lower in the FCC group in previous studies [41, 42]. These results indicate that allowing parents to visit their infants and perform SSC with adequate hygiene practice is safe, and it could be applied in Chinese NICUs.

According to the definition of KC, the mother is the optimal provider for SSC, to promote breastfeeding. However, in China, women are encouraged to stay at home and to rest completely for 1 month after birth (called “doing the month”) for recovery [43]. These traditional postpartum practices are accepted regardless of a woman’s age and education [44]. This resting period results in mothers not being able to perform even one or a few hours’ session of SSC per day after discharge from the hospital, which will greatly delay mother-infant exposure in the first month. In this case, father-infant SSC is an optimal alternative.

Whereas most studies focus on the effects of maternal-infant SSC, few studies have researched the father’s participation in SSC. Ludington-Hoe et al. and Erlandsson et al. found that infants who received father-infant SSC maintained higher skin temperature and had better state behavior responses [45, 46]. In addition, no negative effects were observed on the metabolic rate and energy balance in performing paternal SSC [47, 48]. Evidence also suggested that, to some extent, father-infant SSC was capable of decreasing pain response in preterm infants [49–51]. Infants who received KC cried less, and fathers communicated more vocally not only with the newborn but with the mother. Fathers who provided SSC were more willing to be involved in infant care [52], which established bonding and attachment [53] with the infant and created a more stimulating, more harmonious, and generally better family environment [54] beneficial for infant development [23]. Qualitative studies also reported that fathers felt grateful for being needed; they also felt more included in the process, which facilitated their attainment of a paternal role and achievement of equal parenthood [55–58].

As mentioned previously, premature birth can be stressful and traumatic for parents and can cause negative consequences for the natural establishment of the parent-infant relationship. The NICU environment interrupts parental involvement in caring for the infant and jeopardizes the process of attachment between parents and infant. It is recognized that fathers play an important role in the care of their children. Positive father-child interactions established at an early age have been shown to reduce cognitive delay in infants [59]. A father’s involvement is associated with improved cognitive outcomes in preterm infants, which may suggest a possible intervention [60]. Studies show that fathers of preterm infants often feel stressed [61], overwhelmed, isolated, and out of control [62]. Parental psychological well-being and parent-child interaction may affect infants’ development outcomes. However, these negative feelings appeared to be relieved by offering SSC, as fathers reported feeling in control and at ease when they were more involved in caring for their infants. By providing SSC, fathers consider themselves an important part in the course of caring for infants through physical closeness [55].

The aim of the present study is to investigate whether paternal SSC is safe and how it affects outcomes for moderately preterm infants born at GA 32^0^–34^6^ weeks. We hypothesize that the practice of father-infant SSC in the NICU is feasible and will benefit both infants’ and fathers’ well-being.

## Objectives

### Primary objective

The primary objective of this trial is to study the effect of early paternal SSC versus traditional care (TC) on neurodevelopmental outcomes measured by salivary cortisol, the PIPP, the Infant Neurological International Battery (INFANIB), and neuroimaging.

### Secondary objectivesThe secondary objectives of the study are the following:

- To assess the safety and physiological stability of father-infant SSC
- To study the effect of father-infant SSC on infants’ state behaviors
- To investigate the effect of father-infant SSC on paternal psychological status
- To investigate the effect of father-infant SSC on father-infant attachment

## Methods

### Study design

**Fig. 1:**
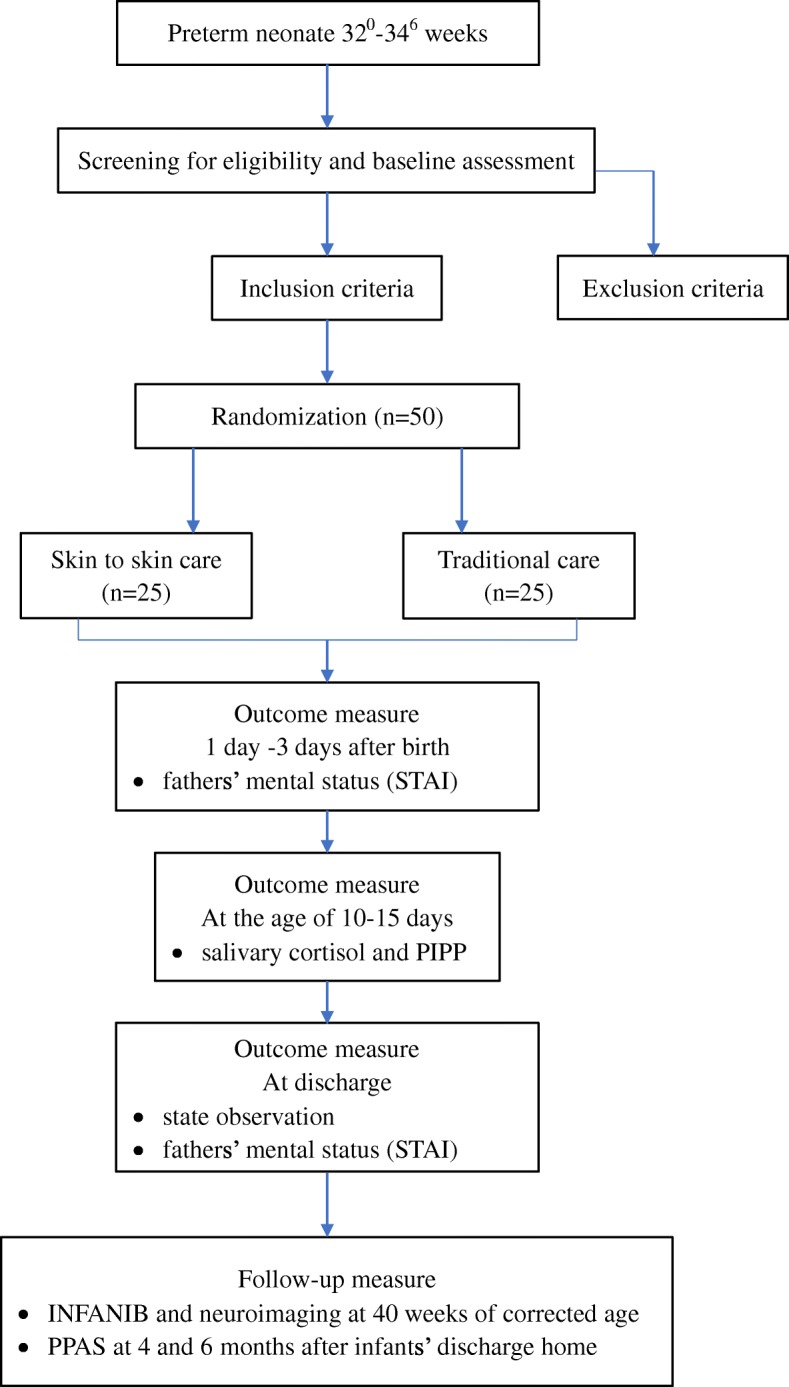
Flowchart of study design

This study is a randomized controlled, parallel-designed clinical trial. The infants in one group will receive 1-h paternal SSC, and the infants in the other group will receive no paternal SSC. We will recruit singleton and twin preterm infants with a GA of 32^0^–34^6^ weeks and with BW ≥ 1500 g delivered either vaginally or by C-section. Infants with unstable medical condition who are evaluated by a neonatal doctor will be excluded. The infants allocated to the TC group will receive standard care in an incubator during hospitalization. The infants in the intervention group will be offered father-infant SSC at least 1 h every day in addition to standard care. Further treatment during hospitalization will not differ between the groups. The flowchart for the study is shown in Fig. 1. The schedule of study enrollment, interventions, and assessments is given in Table 1.

**Table 1:** Schedule of enrollment, interventions, and assessments

Time point	Study Period								
	Enrolment	Allocation	Post-allocation						
	*-t*_*1*_		*t*_*1*_	*t*_*2*_	*t*_*3*_	*t*_*4*_	*t*_*5*_	*t*_*6*_	*t*_*7*_
Enrolment:									
Eligibility screen	X								
Informed consent	X								
Randomization and allocation		X							
Interventions:									
SSC group			X						
TC group			X						
Assessments:									
State-Trait Anxiety Inventory, STAI				X		X			
Salivary cortisol					X				
Brazelton Neonatal Assessment Scale, BNAS						X			
Neuroimaging							X		
Infant Neurological International Battery, INFANIB							X		
Paternal Postnatal Attachment Scale, PPAS								X	X

t_1_ = During hospitalization

t_2_ = 1 day to 3 days after birth

t_3_ = 10-15 days after birth

t_4_ = 37 weeks of corrected age or before discharge

t_5_ = 38-42 weeks of corrected age

t_6_ = 4 months after discharge

t_7_ = 6 months after discharge

### Study setting and location

This study is currently being conducted at a level 3 NICU at the Women’s Hospital School of Medicine Zhejiang University in China, where more than 300 LBW infants are being treated per year. A total of 105 beds are available in the NICU wards, with more than 100 pediatricians and nurses.

### Study population

All the infants will be delivered either vaginally or by C-section in the hospital. Infants with GA ≥ 31 weeks and/or with BW 1500–2300 g will be transferred to the NICU. Male and female healthy preterm infants will be selected from the NICU according to the inclusion criteria.

### Patient adherence

To ensure participant adherence, the investigator will maintain close contact with the fathers in the SSC group and coordinate the intervention time with fathers at their convenience. Fathers may withdraw from the trial for any reason at any time. Fathers who commit to participation but miss several sessions of SSC because of work or time limitations will be allowed.

### Inclusion criteria:

1. GA between 32^0^ and 34^6^ weeks with BW ≥ 1500 g
2. Apgar scores at 1 min and 5 min ≥ 7
3. Vaginal or caesarean delivery
4. Medically stable for 24–72 h (definition of stability is based on the infant’s vital signs, with temperature ranging from 36.5 to 37.5 °C, SpO_2_ > 90%, respiratory rate < 60/min, heart rate of 100 beats/min to 180 beats/min)

### Exclusion criteria:

1. Congenital anomalies
2. Metabolic diseases
3. Severe periventricular/intraventricular hemorrhage (IVH, Grades III–IV)
4. History of minor or major surgery
5. Hospitalization duration of less than 2 weeks
6. Infants belonging to single-parent families
7. Infants with intubation and mechanical ventilation

### Trial treatments

All infants admitted to the NICU will be given parenteral nutrition, cared for under radiant warmers, and treated with further interventions according to the health status of the infants. After the initial stabilization, infants will be randomly assigned to either the SSC group or the TC group according to randomization and allocation rules.

### Intervention group

Infants in the intervention group will be offered SSC as soon as they are stable (approved by a pediatrician in the unit), and they are expected to receive SSC every day during hospitalization. SSC will be performed between 4:00 pm and 6:00 pm for at least 1 h in a private room adjacent to the unit with an ambient temperature between 22 and 24 °C and appropriate light and sound levels. Fathers will wear a comfortable gown and be seated on a reclining chair at an angle of 40 to 60°. Each infant will be taken out of the incubator, unclothed (only diaper and cap are kept on), and placed in an upright position on the father’s chest. The infants will be covered by a blanket to keep them warm. Fathers will be educated on how to hold the infant correctly and encouraged to watch their child and talk to them. Father and baby will be left in the kangaroo position for at least 1 h. During SSC, infants will remain attached to a cardiorespiratory monitor and will be observed by a nurse; routine nursing including parenteral nutrition administration and IV injection can also be performed. Because SSC is not routine care in our hospital, this practice will follow the World Health Orgainization (WHO) guidelines [63].

If hypothermia or apnea of any duration accompanied by desaturation occurs during the SSC, the intervention will be discontinued, and the eligibility of the infant will be reassessed. Infants in the intervention group will receive the same standard treatment as infants in the TC group except for at least 1 h of SSC every day.

### Control group

Conversely, infants in the TC group will be cared for under radiant warmers or in the incubators and treated with routine nursing procedures. These infants will not receive any paternal SSC during hospitalization.

## Outcome measures

### Primary outcome measure

The primary outcome is the neurodevelopmental outcomes of preterm infants, which will be measured by salivary cortisol and PIPP during hospitalization (at the age of 10–15 days) and by INFANIB and neuroimaging at term-equivalent age (40 weeks of corrected age).

#### Infants’ salivary cortisol

Salivary samples will be collected twice, before (baseline) and after (response) a heel stick, which is part of routine care. One hour before the heel stick, the infants will not be fed. One sample will be collected just before the heel stick, and the other will be collected 20 to 30 min after completion of the blood draw. Saliva is collected using a Salivette® Cortisol (Sarstedt, Numbrecht, Germany). After collection, samples will be centrifuged at 4000 rpm for 15 min. Afterward, they will be frozen at − 40 °C until analysis. All the samples will be safely stored in the laboratory of the hospital. Salivary cortisol levels will be determined using the quantitative cortisol enzyme-linked immunosorbent assay (ELISA) Kit (R&D Systems, Inc. Minneapolois, MN, USA), which is specifically designed for research purposes with a high sensitivity of 0.111 ng/mL, which allows the measurement of low concentrations of cortisol. Samples will be run in duplicate, and all samples from each individual will be run in the same assay.

#### Premature Infant Pain Profile

The PIPP is a pain scale originally developed to assess procedural pain in preterm neonates [64]. This profile consists of seven items, including three behavioral indicators (brow bulge, eye squeeze, and nasolabial furrow), two physiological indicators (heart rate and oxygen saturation), and two contextual indicators (GA and behavioral state). Each indicator is evaluated on a 4-point Likert-type scale. Total scores range from 0 to 21, reflecting minimal to severe pain level. The construct validity and internal consistency of the PIPP have been well established [65]. Behavioral indicators will be recorded by camera, and physiological indicators will be collected via pulse oximeter. The infants’ GA will be retrieved from the medical records.

All digital video files will be independently analyzed by two researchers after they are mixed to ensure data blindness.

#### Infant Neurological International Battery

The INFANIB is used as a screening tool to assess infants’ neurodevelopmental status at term-equivalent age (40 weeks of corrected age). This instrument was established by Ellison and Browning in 1985 and was designed to detect gross motor developmental delay and, it was hoped, to predict later neurological dysfunction during the first 18 months of life [66]. Evidence shows that the INFANIB is an appropriate neurological outcome test for premature and/or LBW infants [67–69].

#### Magnetic Resonance Imaging (MRI)

Quantitative MRI will be used to observe preterm infants’ brain growth at term-equivalent age. Total cerebral volume, volumes of white and gray matter, and the cerebral cortex will be measured to predict brain development in preterm infants.

4.1.4.1. MRI data acquisition

MRI examinations will be performed with a 1.5 T magnet (Siemens Medical Systems, Berlin, Germany) equipped with an eight-channel phased array head coil. The following sequences will be acquired for all subjects: (1) standardized T1-weighted and T2-weighted images; (2) magnetization prepared rapid acquisition gradient echo (MPRAGE) images obtained using the following parameters: 144 slices, TR = 2300 ms, TE = 3.39 ms, slice thickness = 1 mm, flip angle = 7°, inversion time = 1100 ms, FOV = 200 × 256 mm^2^, and in-plane resolution = 200 × 256; (3) blood oxygen level-dependent (BOLD) images obtained using the following parameters: 24 axial slices, thickness/gap = 5.0/0 mm, in-plane resolution = 64 × 64, TR = 2000 ms, TE = 30 ms, flip angle = 90°, and FOV = 210 × 210 mm^2^.

Pulse oximetry and heart rate will be monitored throughout the intervention. Ear protection will be used for each infant (MACK’S Earplugs, McKeon Products, Inc., Warren, MI, USA; MiniMuffs, Natus Medical, San Carlos, CA, USA). Chloral hydrate (25–50 mg/kg) will be administered to infants.

### Secondary outcomes

#### Infants’ safety and physiological stability

Safety will be measured by the incidence of hypothermia (< 36.5 °C) and apnea of any duration accompanied by desaturation (SpO_2_ < 88% in room air). Infants’ physiological stability, such as respiratory rate (respiratory rate < 60/min), heart rate (100 beats/min to 180 beats/min), and oxygen saturation (SpO_2_ > 90%) variables, will be recorded during SSC and throughout hospitalization.

#### Infant’s state observation at discharge

The Neonatal Behavioral Assessment Scale (NBAS) was developed by Dr. Brazelton to examine infant behavioral capacity [70]. According to the NBAS, items are selected to assess infants’ state regulation before hospital discharge. States are defined as follows: *Quiet sleep* - deep sleep with regular breathing, eyes closed, no spontaneous activity; *Active sleep* - light sleep with eyes closed, rapid eye movements can be observed, low activity level; *Sleep-wake transition* - drowsy or semidozing, eyes may be open, dull and heavy lidded, even closed, activity level variable, reactive to sensory stimuli, but response is delayed, state change after stimulation frequently noted, movements are usually smooth, dazed look when the infant is not processing information and is not fully alert; *Unfocused wakefulness* - alert, with a bright look, the infant seems to focus attention on source of stimulation, motor activity is at a minimum, a kind of glazed look exists, which can be easily broken through; *Alert wakefulness* - eyes open, considerable motor activity with movements of the extremities, reactive to external stimulation with startles and motor activity; *Fuss/cry* - intense crying, which is difficult to break through with stimulation, motor activity is high, the typical crying face with cupped tongue should be seen.

Observations will take place during the same time period (12:30–13:30 pm) by one trained researcher. The researcher will note the predominant state expressions during the first minute of every 10-min period throughout the hour of observation.

#### Fathers’ mental status

The State-Trait Anxiety Inventory (STAI) will be used to assess fathers’ mental status in regard to state and trait anxiety [71]. The questionnaire consists of two parts, each containing 20 statements. One part assesses state anxiety, and the other part assesses trait anxiety. This questionnaire was translated into a Chinese version in 1988, and the internal consistency reliability was 0.95 [72, 73]. All items are scored according to a 4-point Likert scale. Two parts are calculated, with a minimum score of 20 and a maximum score of 80. Higher scores indicate higher levels of anxiety.

Fathers will complete this questionnaire at the following time points: (1) 1 day to 3 days after the infant’s birth and (2) when the infant is discharged from the NICU.

#### Father-infant attachment

Father-infant attachment will be evaluated by the use of the Paternal Postnatal Attachment Scale (PPAS) originally designed by John T. Condon and colleagues [74]. This is a 19-item, self-report questionnaire including three subscales: patience and tolerance, pleasure in interaction, and affection and pride. The reliability and construct validity of the instrument has been well established, with the Cronbach’s alpha ranging from 0.7–0.8 [75]. Consent for using this questionnaire was obtained from the original authors. The original PPAS has been translated into Chinese and back-translated into English, and it was revised to check for any difference between the two versions. The reliability of the Chinese version scale was 0.809.

Fathers will receive the online PPAS at 4 and 6 months after discharge from the NICU to assess the father-infant relationship.

### Adverse events

This study will be implemented under thorough assessment and close monitoring by the medical staff. Although negative effects are rare, there is a chance that infants may have hypothermia or hypoglycemia or experience physiological instability during SSC. If negative effects occur, SSC will be discontinued, and the infants will be reassessed by medical staff.

### Sample size

The sample size is calculated based on the results from Xiaomei Cong [19] on the level of salivary cortisol of study 2, KCH (kangaroo care heel stick study condition): mean (M) = 0.21, standard deviation (SD) = 0.12; IH (incubator heel stick study condition): M = 0.57, SD = 0.61. We estimate that a sample size of 25 participants per group (50 in total) would be sufficient to detect a difference between the experimental group and the control group, assuming a two-tailed test for a type I error of 5% and a type II error of 20%. This sample size is calculated using the G*Power 3.1.9.2 software. Additional file 1 details the sample size calculation.

### Recruitment

Infants admitted to the NICU ward of the Women’s Hospital School of Medicine Zhejiang University, where more than 300 LBW infants are being treated per year, are eligible. Recruitment is being conducted between September 2017 and April 2019.

### Assignment of interventions

#### Allocation

A simple random sampling method is used. Fifty numbers are generated from a list of randomization codes by a medical staff member who is not involved in the experiment. The numbers are ordered from small to large; the first 25 numbers are set to be the intervention group, and the remaining numbers are set to be the control group. Fifty numbers are mixed and then put into opaque, sealed envelopes. When a preterm infant meets the inclusion criteria, he or she is assigned to either the intervention group or the control group according to the randomly generated number by a medical staff member who is not involved in the experiment.

### Data collection, management, and analysis

#### Data collection

Two researchers will collect the data, which will be kept in a locked cabinet in the NICU. The data are accessible only by the researchers. The following clinical data will be collected: (1) parents’ and infants’ demographics, collected from the medical record; (2) infants’ condition during hospitalization: daily weight, head circumference, temperature, oxygen saturation and heart rate, total amount of enteral and parenteral nutrition given, any sepsis or infection, NEC, duration of antibiotic, biomedical, ultrasound, X-ray and any other test results, any adverse events; (3) observation of SSC: time and frequency of SSC, any cause for interruption or termination of SSC, adverse events; (4) the online STAI and PPAS questionnaires, sent to the fathers’ telephones, with the completed data collected and securely stored by the SoJump online survey software.

#### Biological specimen management

The infants’ saliva will be collected, and samples will be transferred to the laboratory. The saliva will be centrifuged at 4000 rpm for 15 min. Afterward, the samples will be safely stored at − 40 °C until analysis. A radioimmunoassay for cortisol will be used to analyze cortisol concentrations in the saliva. No additional specimens will be stored, and all samples will be discarded appropriately.

### Data analysis

The data analysis will be conducted on an intention-to-treat basis. All the study subjects will be analyzed in the groups into which they were originally randomized.

Descriptive characteristics will be analyzed using frequencies and percentages. The independent *t* test and a repeated measures analysis of covariance (RM-ANCOVA) will be used to compare differences between the two groups for continuous variables. Categorical variables will be compared using the Pearson chi-square test or Fisher’s exact test. *P* values less than 0.05 will be considered significant. Data will be analyzed using the statistical software SPSS 23.0 (IBM SPSS Statistics, IBM Corporation, Armonk, NY, USA).

## Discussion

The design, outcome measures, sample size calculations, and procedures of this study protocol on paternal-infant skin-to-skin contact (SSC) for moderately preterm infants are in accordance with the Standard Protocol Items: Recommendations for Interventional Trials (SPIRIT) 2013 statement for clinical trial protocols (see Additional file 2).

KC is recognized worldwide as an evidence-based care method for preterm and/or LBW infants. The key component of KC is SSC between the baby and the mother. To improve preterm birth outcomes, the WHO recommends that KC should be provided as routine care for newborns weighing 2000 g or less at birth; the WHO also recommends that KC should be initiated in health care facilities as soon as the newborns are clinically stable [17]. Among 10 countries with the greatest number of preterm births, China ranks second; in China, approximately 1,172,300 preterm infants were born in 2010 [2]. However, practices in the NICU vary from province to province, and they depend on differences in economic status. Hence, practice is a factor that may have an essential influence on the scale-up and adoption of KC in China.

Generally, discharging vaginal and cesarean birth mothers on the third and seventh postpartum day, respectively, is the standard procedure. For vaginal birth mothers before discharge, performing KC in the NICU is still feasible if the mother is in good health. For cesarean births, mothers are not prepared to offer KC because of the surgical incision on their abdomen. Furthermore, women are encouraged to rest indoors for a month (“doing the month”) after giving birth to help facilitate postpartum recovery to improve future health and prevent diseases. Although “doing the month” has some negative effects, many Chinese women adhere to this conventional practice regardless of age and education [43, 44, 76]. Consequently, implementing early and continuous KC for preterm infants in the NICU encounters additional setbacks after the mother is discharged.

However, fathers can also perform KC for infants, whether in the hospital or after discharge. According to the definition of KC, the mother is the optimal provider of KC because SSC between mother and infant is the basis of early successful and exclusive breastfeeding [77, 78]. Nevertheless, the father and even other family members are suggested to perform KC while the mother is not available. Evidence shows that the father plays a significant role in infant care. Findings from previous studies show that fathers who provide SSC induce pain alleviation, paternal-infant attachment, and family bonding and create a more harmonious caregiving environment [55, 57, 79, 80], which can be beneficial to infants’ development. However, studies on fathers providing SSC remain limited in China; thus, the effects of paternal SSC on both preterm infants and fathers have not been well studied.

This study aims to investigate the safety and effects of paternal SSC on neurodevelopmental outcomes in preterm infants. The data gathered in this study can be used to promote the implementation of early paternal SSC in the NICU and modify guidelines and procedures to facilitate the involvement of fathers in caring for their preterm babies when mothers are absent.

### Trial status

The study is currently recruiting participants, and the first participant was recruited in September 2017. The official retrospective registration number of the study is ChiCTR-IOR-17012745. The entire study is expected to be completed by the end of December 2019.

## Additional files

Additional file 1: Sample size calculation. (DOCX 193 kb)

Additional file 2: SPIRIT 2013 checklist: recommended items to address in a clinical trial protocol and related documents. (PDF 588 kb)

Additional file 3: Informed consent form. (DOCX 139 kb)

## Abbreviations

BW: Birth weight
GA: Gestational age
KC: Kangaroo care
LBW: Low birth weight
MRI: Magnetic resonance imaging
NBAS: Neonatal Behavioral Assessment Scale
NEC: Necrotizing enterocolitis
NICU: Neonatal intensive care unit
PIPP: Premature Infant Pain Profile
PPAS: Paternal Postnatal Attachment Scale
SSC: Skin-to-skin contact
STAI: State-Trait Anxiety Inventory
