# Supplementary figures and images for: Early father-infant skin-to-skin contact and its effect on the neurodevelopmental outcomes of moderately preterm infants in China: study protocol for a randomized controlled trial
Source: Trials. 2018 Dec 22;19:701. doi: 10.1186/s13063-018-3060-2 (PMC6303962; doi:10.1186/s13063-018-3060-2)

Sample Size Calculation


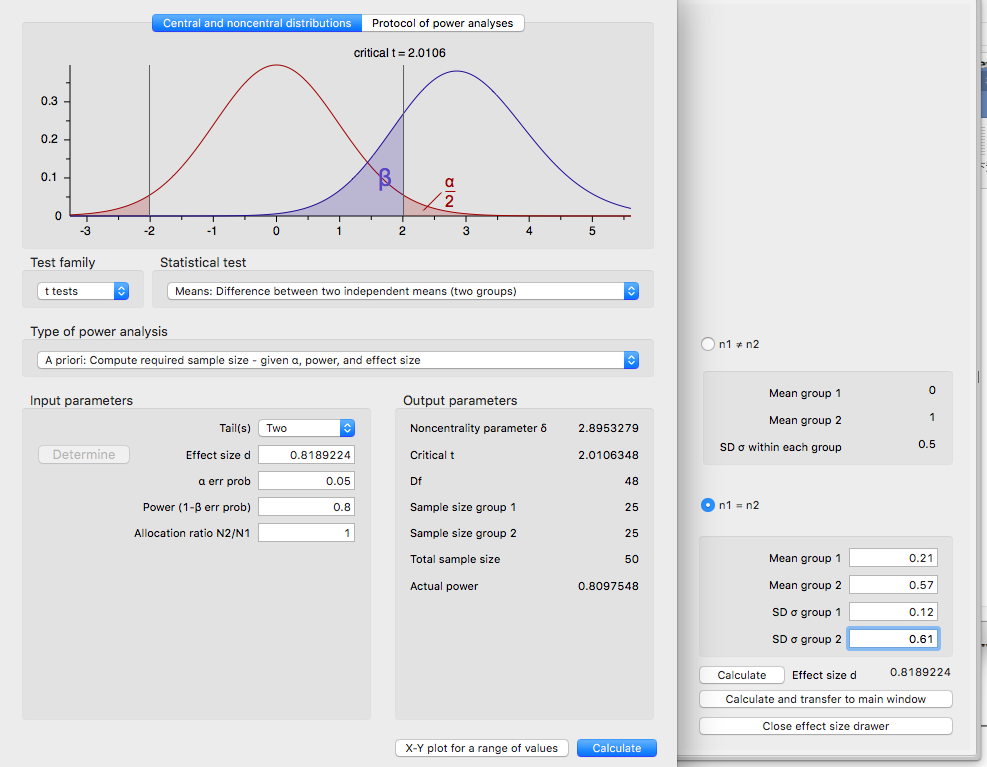

Supplement: Supplementary file 1 — Sample size calculation. (DOCX 193 kb) [file 13063_2018_3060_MOESM1_ESM.docx]
