# Supplementary material for: Early father-infant skin-to-skin contact and its effect on the neurodevelopmental outcomes of moderately preterm infants in China: study protocol for a randomized controlled trial
Source: Trials. 2018 Dec 22;19:701. doi: 10.1186/s13063-018-3060-2 (PMC6303962; doi:10.1186/s13063-018-3060-2)
Supplement: Supplementary file 3 — Informed consent form. (DOCX 139 kb) [file 13063_2018_3060_MOESM3_ESM.docx]

**Informed Consent·Inform Page**

Dear patient,

Your baby is diagnosed as preterm infant. We sincerely invite you and your baby to participate a project, named “Early father–infant skin–to–skin contact and its effect on the neurodevelopment outcomes of moderately preterm infants in China: a randomized controlled trial” which is funded by Natural Science Foundation of Zhejiang Province (CN) (Project Number: Y17H040025). This study has been approved by Women's Hospital School of Medicine Zhejiang University Medical Ethics Committee.

Before making your decision on whether participating in this study or not, please read the following materials thoroughly. It can help you understand the purpose, procedure and timeline of the study. It also helps you aware of the benefits, risks, and discomforts after attending the study. If you wish, you can discuss with your relatives or friends, or ask the doctor to give you an explanation to help you make a decision.

1. Background and objective of the study

1.1 Background

Every year, an estimated 15 million babies are born preterm (before 37 completed weeks of gestation), more than 60% of preterm births occur in Africa and South Asia, accounting 11.1% of the total births in the world. Premature infants are likely to suffer from brain damage in varying degrees and may cause cerebral palsy due to inadequate gestational age and malnutrition in early life, which can lead to brain damage and dysfunction. In our country the incidence of mental retardation on premature infants is as high as 7.8%, not only affects the development of the intellectual quality of the next generation, but also brings great mental and economic burden to individuals, families and society.

Kangaroo care is an intervention based on early, prolonged, and continuous skin to skin contact (SSC) between infant and mother (or father if mother is not available). Kangaroo care is good for improving preterm infants’ outcomes, including the stabilizing vital signs (body temperature, breathing, rhythm), reducing crying, maintaining good sleep, promoting brain development, and helping parents build confidence in caring for their children.

1.2 Objective

The aim of this study is to implement early father–infant SSC in the NICU wards, observing its safety and effects of neuro–protection on moderate preterm infants. Our thought is that father–infant SSC is feasible and necessary once infants reach medically stable. We predict that early father–infant SSC could benefit infants’ outcomes as well as having positive effect on fathers’ mental health.

1.3 Institution of recruitment and the number of predicted included participants

Institution of recruitment is Women's Hospital School of Medicine Zhejiang University. The number of predicted included participants is sixty.

2. Inclusion criteria

a) GA between 32^0^–34^6^ weeks with BW ≥ 1500 g

b) Apgar scores at 1 min and 5 min ≥ 7

c) vaginal and caesarean delivery

d) medically stable for more than 24 h (Definition of stability is based on infant’s vital signs with temperature 36.5–37.5℃, SpO_2_ > 90%, respiratory rate < 60/min, heart rate 100 beats/min –180 beats/min)

3. What will you need to do for the study

3.1 Before you and your baby are eligible to participate in the study, researcher will review the medical records and assess the premature infant. If you and your baby are eligible, you may involve in the study voluntarily and sign the informed consent. If you reject to participate, we will offer routine treatment for you and your baby.

3.2 If you decide to participate in the study, the procedures are as follows:

If you are in the intervention group, you need to provide SSC for your baby at least 1 hour every day as soon as your baby is clinically stable, lasting for 14 days or longer while your baby is hospitalized. During hospitalization, we will collect infant’s data including:

a) general information (vital signs, weight, head circumference, et al).

b) a video of routine heel stick, analyzed for infant’s physiological and behavioral reactions, and salivary samples measured for cortisol levels.

c) observation of state behaviors measured by Neonatal Behavioral Assessment Scale (NBAS) at 37 weeks of corrected age or before hospital discharge.

After the baby is discharged, you need to take your baby to follow–up visit at 40 weeks of corrected age. Infant will be assessed by neuroimaging and Infant Neurological International Battery (INFANIB)

Father needs to complete the State–Trait Anxiety Inventory at the time when infant is admitted when infant is discharged. After 4 and 6 months infant’s discharge, father need to complete the Paternal Postnatal Attachment Scale.

4. Benefits from the study

For the infant: SSC can maintain physiological stability, decrease pain response and lower cortisol level, regulate sleep patterns, decrease mortality and morbidity. Although it is unclear whether SSC has long lasting effects on preterm infant as regard to neurodevelopmental outcomes, studies show that kangaroo care might have a direct impact on infant neurophysiological organization and early SSC plays an important role in the maturation of the autonomic and circadian systems in preterm infants. Long–term follow–up studies explore that kangaroo care preterm infants show attenuated stress response, have organized sleep and better cognitive control, maintain long–lasting social and behavioral protective effects even after 10 and 20 years.

For the father: feel grateful, in needed and feel more included which facilitate them attain paternal role and achieve more equal parenthood. Willing to involve in baby care which on the one hand, establish bonding attachment relationship with infant and on the other hand, help create a more stimulating and harmony context and better family environment where it is beneficial for infant development.

Despite evidence has shown that SSC is beneficial for preterm infants, this cannot guarantee effective to you and your baby. Besides, SSC is not the only intervention for you and your baby. You can discuss with the medical professionals and researchers for an alternative intervention.

5. Possible risks, side–effects, discomforts and inconvenience from the study

This study will be implemented under thoroughly assessment and close monitoring by the medical staff. Although negative effects are rare, there are chances that infants may have hypothermia, hypoglycemia or suffer from physiological instability during SSC. Once negative effects happen, SSC will be discontinued and infants will be re–assessed by medical staff.

6. Related expenses

Related expenses for SSC are needed according to hospital policy if you decided to participate in the study.

7. Personal information confidentiality

You and your infant medical records (medical records, laboratory research /CRF etc.) will be kept intact. The researchers, ethics committee and drug supervision and management departments will be allowed access to your medical records. Any of the results from this study for public report will not disclose your personal identity. We will make every effort to protect your personal medical information privacy within the law.

In accordance with the ethics of medical research, the test data will be available for public inquiry and sharing except for personal information. Query and sharing will be limited to electronic database based on network, which guarantee not any personal information is disclosed.

8. Access for more information

You can ask any questions about the study at any time, and obtain corresponding reply.

If there is any important new information in the course of the study, which may affect your willingness to continue to participate in the study, researcher will inform you.

9. Participate in or withdraw from the study

Decision is made completely voluntarily. You can reject to participate in the study or withdraw from the study at any time which will not affect the relationship between you and medical staff, and no harm will be done to you and your infant’s treatment or other benefits.

Doctors or researcher may discontinue SSC taking into consideration of your maximum benefit.

10. What do you need to do now?

Decision is made completely by yourself (and your family).

Before making any decision, please ensure that you have enquired about related questions.

Thank you for reading the above materials. If you decided to participate in the study, please let the researchers know who will arrange everything for you about the study. Please save this consent.

**Informed Consent·Sign Page**

Name of the project: Early Father–Infant Skin to Skin Contact and the impact on Neurodevelopment Outcomes of Late Preterm Infants in China

Project committed institution unit: Women's Hospital School of Medicine Zhejiang University

Patient Statement

I have read and understood adequately the descriptions, which the doctor explained to me. I am aware of the possible risks and benefits from the study. I understand that it is voluntary to participate in the study. I am sure I have thought this through and knowing that:

- I can enquiry for more information at any time.
- I can withdraw from the study at any time without discrimination or revenge and my medical treatment and right will not be affected.

I agree that ethics committee can access to my medical records.

I will receive a signed and dated copy of the informed consent.

Finally, I decide to participate in the study and promise that I follow the researchers’ orders as much as possible.

Signature of patient: __________________

Date of signing: _____________________

Contact number: _____________________

Researcher Declaration: I have fully explained the nature, risks and benefits of SSC to the above patient/relative/guardian, and answered their questions. To the best of my knowledge, the patient/relative/guardian has been informed adequately and has consented. The medical staff will do their utmost to perform emergency management for the patient if any above stated description occurs.

Researcher’s signature: __________________

Date of signing: ________________________

In the event of inconsistency or discrepancy between the Chinese version and the English version, the Chinese language version shall prevail.
